# Pregnancy and the Risk of Autoimmune Disease

**DOI:** 10.1371/journal.pone.0019658

**Published:** 2011-05-18

**Authors:** Ali S. Khashan, Louise C. Kenny, Thomas M. Laursen, Uzma Mahmood, Preben B. Mortensen, Tine B. Henriksen, Keelin O'Donoghue

**Affiliations:** 1 Anu Research Centre, Department of Obstetrics and Gynaecology, Cork University Maternity Hospital, University College Cork, Wilton, Cork, Republic of Ireland; 2 National Centre for Register-Based Research, University of Aarhus, Aarhus, Denmark; 3 Perinatal Epidemiology Research Unit, Department of Paediatrics, Aarhus University Hospital, Aarhus, Denmark; Institute Biomedical Research August Pi Sunyer (IDIBAPS) - Hospital Clinic of Barcelona, Spain

## Abstract

Autoimmune diseases (AID) predominantly affect women of reproductive age. While basic molecular studies have implicated persisting fetal cells in the mother in some AID, supportive epidemiological evidence is limited. We investigated the effect of vaginal delivery, caesarean section (CS) and induced abortion on the risk of subsequent maternal AID. Using the Danish Civil Registration System (CRS) we identified women who were born between 1960 and1992. We performed data linkage between the CRS other Danish national registers to identify women who had a pregnancy and those who developed AID. Women were categorised into 4 groups; nulligravida (control group), women who had 1st child by vaginal delivery, whose 1st delivery was by CS and who had abortions. Log-linear Poisson regression with person-years was used for data analysis adjusting for several potential confounders. There were 1,035,639 women aged >14 years and 25,570 developed AID: 43.4% nulligravida, 44.3% had their first pregnancy delivered vaginally, 7.6% CS and 4.1% abortions. The risk of AID was significantly higher in the 1st year after vaginal delivery (RR = 1.1[1.0, 1.2]) and CS (RR = 1.3[1.1, 1.5]) but significantly lower in the 1st year following abortion (RR = 0.7[0.6, 0.9]). These results suggest an association between pregnancy and the risk of subsequent maternal AID. Increased risks of AID after CS may be explained by amplified fetal cell traffic at delivery, while decreased risks after abortion may be due to the transfer of more primitive fetal stem cells. The increased risk of AID in the first year after delivery may also be related to greater testing during pregnancy.

## Introduction

Autoimmune diseases (AID) are most common among women and increase in incidence following their reproductive years [1]. AID are caused by an immune reaction against self–antigens due to disturbances in T or B cell regulation or function, and autoimmunity may occur in a genetically susceptible individual if an antigen is inadvertently targeted by T or B cells potentially due to environmental or other factors triggering a break in tolerance [2]. Those autoimmune diseases more common in women include systemic lupus erythematosus (SLE; 9∶1), autoimmune thyroid disease (8∶1), scleroderma (5∶1), rheumatoid arthritis (4∶1) and multiple sclerosis (3∶1), while type 1 diabetes and inflammatory bowel diseases have almost the same female to male ratios of 1∶1 and primary sclerosing cholangitis is more prevalent in men.

The human immune system shows some degree of sexual dimorphism. In general, women have higher CD4 cell counts than men, which contributes to an increased CD4/CD8 ratio [3], higher levels of plasma IgM and greater Th1 cytokine production [4]. Sex hormones have been implicated in autoimmunity due to their capacity to modulate the immune response via androgen and estrogen receptors [2]. Dramatic changes in the levels of estrogens, progesterone and other hormones such as cortisol also occur during pregnancy [1]. However, it is unlikely that the difference in sex hormones between women and men alone explains the preponderance of AID in women; most AID occur after reproductive years and administration of sex steroids does not have disease-altering effects [2].

Whether pregnancy or parity influence the development of AID remains a subject of much debate [1], [2]. Since pregnancy involves complex interactions between hormonal and immunological factors, it is plausible that it could have differing effects on the development of AID [1]. While results of published studies on the association between pregnancy and SLE are contradictory [5], [6], evidence suggests that the risk of multiple sclerosis onset is reduced during gestation and increased in the first 6 months after pregnancy [7], [8]. It is unclear, despite much investigation, whether there is an association between parity and the risk of rheumatoid arthritis [9]–[11]. The currently available data do not provide convincing evidence that parity provides an explanation for the female predominance in the majority of autoimmune conditions.

Trafficking of fetal cells into the maternal circulation begins very early in the pregnancy and the effects of this cell traffic are long lasting [12], [13]. All types of fetal cells, including stem cells, cross the placenta during normal pregnancy to enter the maternal blood and tissues, where they may be located decades after pregnancy [14]–[16]. Fetal microchimerism is defined as low levels of fetal cells persisting in maternal tissues during and after pregnancy. The connection between fetal microchimerism and human disease was first made by Nelson, who speculated that microchimerism might be the underlying basis for the higher prevalence of autoimmune disease in women [17]. This hypothesis is supported by the similarities of chronic graft versus host disease (GVHD) to some autoimmune conditions, the prevalence of these diseases in women, their increased incidence after reproductive years, and the fact that GVHD increases with HLA incompatibility of the donor [18]–[20].

Fetal microchimerism has now been investigated in many candidate AID, with some results supporting a role in disease pathogenesis [16], [20], [21]. However, no direct association has been shown between the presence of microchimeric cells and the progression of AID. Reports now suggest that most autoimmune conditions are not significantly associated with more microchimeric cells in blood or tissues when correctly compared to controls. In addition, although fetal cells do appear to accumulate in clinically affected organs, there is no conclusive evidence that fetal cells cause autoimmune disease [20], [22]. Some authors suggest that an explanation for the conflicting results in studies relating fetal microchimerism and autoimmune disease is the migration of fetal cells preferentially into target organs of the disease rather than the circulation. Other explanations for the discrepancies in microchimerism results are differences in experimental designs and the sensitivity and specificity of the techniques used by different investigators [18]. Disease severity might also be a factor, and it may be the tissue damage of AID that leads to recruitment to and colonisation of injury sites by fetal cells, rather than autoimmunity itself [16].

Factors predisposing to the development of fetal microchimerism are much debated. There is more fetomaternal cell trafficking where the placenta is abnormal and in certain complications of the pregnancy such as fetal aneuploidy, pregnancy loss and pre-eclampsia [20], [23], [24]. However, early fetal loss seems the only pregnancy complication significantly influencing the development of microchimerism, and it has been speculated that miscarriage allows more primitive types of fetal cells with the greater capacity to differentiate to enter the maternal circulation [25]. Fetomaternal haemorrhage as a result of first trimester termination of pregnancy has been shown to cause an 80-fold increase in the number of fetal cells in maternal blood [26]. Fetomaternal haemorrhage is also likely to be greater after operative when compared to normal vaginal delivery and thus more microchimerism should be established after caesarean section delivery. It is accepted that even short-term microchimerism can lead to autoimmune disease and the higher amount of trafficking at caesarean section might increase the exposure of these mothers and facilitate the development of autoimmune disease.

The objective of this study was to find out whether risk of new onset autoimmune disease is higher after delivery by caesarean section compared to vaginal delivery and we also aimed to quantify the risk of autoimmune disease after abortion. We investigated the effect of vaginal delivery, caesarean section (CS) and abortion on the risk of subsequent maternal AID using data from the Danish National Registers.

## Methods

### Study cohort

The study cohort consisted of all women born in Denmark between January 1, 1962 and December 31, 1992. The data were obtained by linking the Danish Civil Registration System [27], the Danish National Hospital Register [28] and the Danish Medical Birth Register [29]. The linkage process was performed using the unique personal identification number (CPR-number), which is used in all national registers and enables accurate linkage between all registers [27]. The linkage process enabled us to identify the firstborn children for those women with children, mother and infant place of birth, date of delivery, infant sex, date of migration of any woman who left the country and the woman's date of death if the woman died. From the Medical Birth Register, we obtained data on mode of delivery, induced abortion and date of abortion, single or multiple gestations. From the National Hospital Register we obtained information on AID diagnoses, together with inpatient or outpatient hospital contact. The National Hospital Register includes details of inpatient hospitalization from 1977 and inpatient and outpatient contact from 1995 onward. Date of onset of AID was defined as the date of the first contact with the hospital that led to the diagnosis.

### Methods

Women were grouped in four exposure categories based on their first pregnancy only: 1) Women who had no previous pregnancy; 2) women who had a vaginal delivery; 3) women who had a CS, and 4) women who had an abortion. Abortion was defined as induced abortion before 20 weeks gestation based on International Classification of Disease revisions 8 and 10 (ICD-8: 640, 641, 642 & ICD-10: DO04) [30], [31]. Thirty AID were identified according to ICD-8 and ICD-10 and are listed in Table 1. More details on AID in Denmark are available from recent publications [32].

**Table 1:** International classification of disease: autoimmune disease.

Autoimmune Disease	Categorization		Prevalence per 1000	
	ICD8	ICD10	ICD8/ICD10	ICD10 ONLY
Pernicious Anemia	281.0	D51.0	0.54	0.42
Autoimmune Hemolytic Anemia	283.90–91	D59.1	0.14	0.12
Idiopathic Thrombocytopenic Purpura	446.49	D69.3	0.49	0.48
Thyrotoxicosis	242.00	E05.0	4.99	4.82
Autoimmune Thyroiditis	245.03	E06.3	0.63	0.57
Type 1 Diabetes	249	E10	9.75	9.58
Primary Adrenocortical Insufficiency	255.1	E27.1	0.23	0.20
Multiple Sclerosis	340	G35	2.17	2.04
Guillain Barre Syndrome	354	G61.0	0.60	0.27
Iridocyclitis	364	H20	2.17	1.94
Crohn's Disease	563.01	K50	2.78	2.63
Ulcerative Colitis	563.19	K51	5.65	5.25
Autoimmune Hepatitis	571.93	K73	0.51	0.39
Primary Biliary Cirrhosis	571.90	K74.3	0.16	0.13
Coeliac Disease	269.00	K90.0	0.76	0.70
Pemphigus	694 (×694.05)	L10	0.08	0.07
Pemphigoid	694.05	L12	0.14	0.14
Psoriasis vulgaris	696.09–10, 696.19	L40 (xL40.4)	3.43	2.94
Alopecia Areata	704.00	L63	0.34	0.30
Vitiligo	709.01	L80.9	0.24	0.21
Seropositive Rheumatoid Arthritis	712.19, 712.39, 712.59	M05–M06	6.20	5.82
Juvenile Arthritis	712.09	M08	0.86	0.71
Wegener's Granulomatosis	446.29	M31.3	0.18	0.18
Dermatopolymyositis	716	M33	0.21	0.17
Polymyalgia Rheumatica	446.30–31, 446.39	M31.5–6, M35.3	3.02	2.78
Myasthenia Gravis	733.09	G70.0	0.22	0.19
Systemic Sclerosis	734.0	M34	0.30	0.28
Systemic Lupus Erythematosus	734.19	M32.1, M32.9	0.59	0.53
Sjogren's Syndrome	734.90	M35.0	0.78	0.75
Ankylosing Spondylitis	712.49	M45.9	0.85	0.75

The AID follow-up period was divided into 1) during pregnancy (this category was included for completeness), 2) first year following pregnancy, 3) second year following delivery, 4) third year following delivery, 5) more than three years but less than 10 years following delivery and 6) 10 years or more. This was also applied to the follow-up period following abortion. The follow-up period started in January 1, 1994 and ended in December 31, 2006. Women were followed-up for AID from their 14th birthday until their deaths, migration, onset of AID or end of study period, whichever came first. Women with a diagnosis of AID that included diabetes before the start of the follow-up period were excluded from the study cohort; as diabetes is generally accepted to have a different aetiology than other AID and its onset usually precedes the childbearing years.

### Statistical analysis

Log-linear Poisson regression with aggregated person-years data was used to estimate the relative risk of AID in relation to vaginal delivery, CS and abortions [33]. The models of AID in relation to vaginal delivery and CS were adjusted for age (14–15, 16–17, and in 2 years categories thereafter), calendar year (94–95, 96–97, 98–99, 2000–2001, 2002–2003, 2004–2005, 2006), infant sex (male, female, multiple gestation, no pregnancy) and place of birth (capital city, capital city suburbs, large city, small city, rural area). Models of AID in relation to abortion were adjusted for age, calendar year and place of birth since we had no information on fetal sex in abortions. Age and calendar year were generated as time dependent variables and the other variables were time fixed.

The Poisson models were run separately for vaginal delivery, CS and abortion. The reference group in all the models consisted of women who had no records of pregnancy including abortion. Sensitivity analyses were performed to examine whether age, infant sex or multiple gestation had an effect on association between risk of AID and mode of delivery.

This study was conducted according to the principles expressed in the Declaration of Helsinki. The study was approved by the Danish Data Protection Agency and the Danish National Board of Health. The study was based on secondary data and no individuals were approached, nor did we have access to any other information from the participants. Thus it was not necessary to seek written consent.

## Results

During the study period there were 1,035,639 women in Denmark aged 14 years or more. Of those women 25,570 (2.4%) had a diagnosis of AID during 10,786,229 person-years of follow-up. 459,049 women had their first pregnancy delivered vaginally and 11,439 (2.5%) had an AID. 78,694 women had their first pregnancy delivered by CS, of which 1,787 (2.3%) had an AID, and 186,220 had an abortion in their first pregnancy, of which 4,723 (2.5%) had an AID. 455,214 women had no record of a pregnancy during the study period, of which 11,165 (2.4%) had an AID diagnosis. Of the 186,220 women who had abortions, only 42,682 had no records of other pregnancies, thus the numbers do not add up to 1,035,639. When we excluded women who had records of childbirth and abortions in the study cohort, we had 334,205 women with a normal delivery and 60,000 delivered by CS, while 42,682 had abortions, 455,214 had no pregnancy, and 143,538 had abortions and delivery.

Table 2 presents the incidence of AID in relation to age, calendar year, infant sex, multiple gestation and place of birth. It appears that women who were pregnant had a higher incidence of AID than those who had no pregnancy records and women who had singletons had slightly higher incidence of AID than those who had multiple gestation. It also appeared that the incidence of AID increased with age and with the size of the place of birth.

**Table 2:** Distribution of 25,570 women with autoimmune disease and 10.8 million person-years of follow-up in a cohort of 1 million women residing in Denmark.

Variable	Cases	Person years	Incidence per 100,000 person years
**Infant Sex**			
Female	6289	2300317	273.2
Male	6572	2423568	271.2
Multiple gestation	185	72368	255.6
No children	12524	5989845	209.1
**Age**			
14–19	3007	2100277	143.2
20–25	5021	2295805	218.7
26–29	4377	1694776	258.3
30–35	7188	2612313	275.2
36–41	4816	1669130	288.5
42+	1161	413795.5	280.6
**Calendar year**			
94–95	2941	1413014	208.1
96–97	3258	1502817	216.8
98–99	3723	1588335	234.4
2000–2001	4111	1676766	245.2
2002–2003	4654	1768593	263.1
2004–2005	4760	1866601	255.0
2006	2123	969972	218.9
**Place of birth (mother)**			
Capital city	4585	1728824	265.2
Capital city suburbs	2553	1084167	235.5
Large city	3500	1399005	250.2
Small city	9156	3871445	236.5
Rural area	5776	2702656	213.7

a ‘No children’ includes abortions.

Table 3 presents the relative risk (RR) estimates of AID in relation to normal delivery, CS and abortion. There was no evidence to support an association between risk of AID and vaginal delivery (RR = 0.91, [95% CI: 0.84, 0.99]), CS (RR = 1.02, [95% CI: 0.94, 1.11]) or abortion (RR = 0.97, [95% CI: 0.92, 1.04]) for all follow-up period after delivery. The estimates show that the risk of AID during pregnancy was significantly reduced in relation to vaginal delivery (RR = 0.76, [95% CI: 0.63, 0.93]) and significantly increased in relation to CS (RR = 1.82, [95% CI: 1.39, 2.38]). In contrast, the RR of AID during pregnancy in relation to abortion was close to one and not significantly changed (RR = 0.92, [95% CI: 0.62, 1.37]).

**Table 3:** Adjusted relative risk estimates of maternal autoimmune disease in relation to pregnancy.

Follow-up period	AID and vaginal delivery (N)	Vaginal delivery adjusted RR (95% CI	AID and CS (N)	CS Adjusted RR (95% CI)	AID and abortions (N)	Abortion Adjusted RR (95% CI)
No pregnancy	11165	Reference	11165	Reference	11165	Reference
During first pregnancy	105	0.76(0.63, 0.93)	54	1.82(1.39, 2.38)	25	0.92(0.62, 1.37)
All follow-up period	8206	0.91(0.84, 0.99)	1317	1.02(0.94, 1.11)	1154	0.97(0.92, 1.04)
0 to 11 months	673	1.15(1.03, 1.28)	155	1.30(1.10, 1.55)	78	0.70(0.56, 0.88)
12 to 23	539	0.91(0.81, 1.02)	111	0.99(0.81, 1.20)	104	0.96(0.79, 1.16)
24 to 35	544	0.90(0.81, 1.01)	114	1.08(0.89, 1.31)	93	0.90(0.74, 1.11)
36 to 119	3425	0.84(0.78, 0.92)	546	0.97(0.87, 1.07)	566	1.02(0.94, 1.11
120+	3025	0.95(0.88, 1.04)	391	1.01(0.89, 1.13)	313	1.02(0.91, 1.15)

Vaginal delivery and CS models were adjusted for age, calendar year, infant sex and place of birth. Abortion models were adjusted for age, calendar year and infant place of birth. We had no information about fetal sex or number of babies in abortions. Repeating vaginal delivery and CS models for singletons only did not change the estimates. Negative binomial regression models showed that the Poisson models were not over dispersed.

The risk of AID appeared to be moderately increased in the first year following a pregnancy that ended in vaginal delivery (RR = 1.15, [95% CI: 1.03, 1.28]), and CS (RR = 1.30, [95% CI: 1.10, 1.55]) and moderately reduced in the first year following abortion (RR = 0.70, [95% CI: 0.56, 0.88]). However, the risk of AID was not significantly changed after the first year following delivery apart from a reduction of the risk between year three and year 10 following normal delivery (Table 3).

To examine the effect of age on the observed associations we repeated the Poisson models as described earlier for three age groups: 1) <24 years; 2) ≥24 and <35 years; 3) ≥35 years. The RR estimates of AID were similar in the three age groups following vaginal delivery and abortion. In contrast, the RR of AID in the first year following CS appeared to be higher in the younger (RR = 1.62; [95% CI: 1.03, 2.57]) and older women (RR = 1.61, [95% CI: 0.97, 2.68]). Also, there was a non-significant increase in risk of AID in the second year following CS in the older women (RR = 1.42, [95% CI: 0.87, 2.33]).

We also examined the effect of fetal sex and multiple gestations on the observed estimates. Separate analyses for males, females and singleton pregnancies did not change the results materially.

Tables 4, 5, 6, 7 present the relative risk estimates of specific AID types in relation to vaginal delivery, CS and abortion. There was little evidence to support an association between risk of rheumatoid arthritis (RA) and vaginal delivery (RR = 0.72, [95% CI: 0.55, 0.96]), CS (RR = 1.17, [95% CI: 0.88, 1.55]) or abortion (RR = 0.93, [95% CI: 0.74, 1.18]) during the follow-up period after delivery. A reduction of the risk of RA three to 10 years after vaginal delivery was observed (Table 4). No association was observed between the mode of delivery and Multiple Sclerosis (Table 5). The risk of thyrotoxicosis was increased in the first year after both vaginal delivery (RR 2.15, [95% CI: 172, 2.68]) and CS (RR 1.87, [85% CI: 1.33, 2.64]), but not when the first pregnancy resulted in an abortion (Table 6). Finally the risk of inflammatory bowel disease appeared to be reduced following abortion (RR = 0.86, [95% CI: 0.76, 0.97]), but was not significantly changed after vaginal delivery or CS in the same follow up period (Table 7).

**Table 4:** Adjusted relative risk estimates of maternal Seropositive Rheumatoid Arthritis in relation to pregnancy.

Follow-up period	Cases in vaginal delivery	Vaginal delivery adjusted RR (95% CI)	Cases in CS	CS Adjusted RR (95% CI)	Cases in abortions	Abortion Adjusted RR (95% CI)
No pregnancy (reference)	721	Reference	721	Reference	721	Reference
During pregnancy	6	0.64(0.28 1.43)	1	0.49(0.07, 3.46)	1	0.57(0.09, 4.08)
All follow-up period	758	0.72(0.55, 0.96)	143	1.17(0.88, 1.55)	79	0.93(0.74, 1.18)
0 to 11 months	50	0.82(0.55, 1.21)	10	1.01(0.58, 1.95)	2	0.28(0.07, 1.12)
12 to 23	26	0.41(0.26, 0.67)	12	1.25(0.68, 2.32)	7	0.99(0.47, 2.09)
24 to 35	45	0.70(0.47, 1.05)	10	1.09(0.56, 2.12)	4	0.60(0.22, 1.59)
36 to 119	293	0.64(0.48, 0.87)	59	1.12(0.79, 1.59)	37	0.97(0.69, 1.35)
120+	344	0.86(0.64, 1.16)	52	1.26(0.88, 1.81)	29	1.16(0.79, 1.70)

Vaginal delivery and CS models were adjusted for age, calendar year, infant sex and place of birth. Abortion models were adjusted for age, calendar year, infant place of birth. We had no information about fetal sex or number of babies in abortions.

**Table 5:** Adjusted relative risk estimates of maternal Multiple Sclerosis in relation to pregnancy.

Follow-up period	Cases in vaginal delivery	Vaginal delivery adjusted RR (95% CI)	Cases in CS	CS Adjusted RR (95% CI)	Cases in abortion	Abortion Adjusted RR (95% CI)
No pregnancy (reference)	865	Reference	865	Reference	865	Reference
During pregnancy	8	0.58(0.29, 1.17)	4	1.31(0.49, 3.50)	3	1.51(0.48, 4.68)
All follow-up period	756	1.06(0.82, 1.38)	109	0.93(0.71, 1.22)	118	0.99(0.81, 1.20)
0 to 11 months	47	1.14(0.78, 1.68)	12	1.26(0.69, 2.29)	8	0.94(0.47, 1.88)
12 to 23	25	0.58(0.36, 0.93)	7	0.75(0.35, 1.63)	9	1.01(0.52, 1.95)
24 to 35	46	1.03(0.70, 1.52)	5	0.56(0.23, 1.38)	8	0.90(0.45, 1.81)
36 to 119	333	1.03(0.78, 1.36)	51	0.99(0.71, 1.40)	52	0.94(0.71, 1.24)
120+	305	1.18(0.89, 1.57)	34	0.89(0.60, 1.32)	41	1.08(0.79, 1.50)

Vaginal delivery and CS models were adjusted for age, calendar year, infant sex and place of birth. Abortion models were adjusted for age, calendar year, infant place of birth. We had no information about fetal sex or number of babies in abortions.

**Table 6:** Adjusted relative risk estimates of maternal Thyrotoxicosis in relation to pregnancy.

Follow-up period	Cases in vaginal delivery	Vaginal delivery adjusted RR (95% CI)	Cases in CS	CS Adjusted RR (95% CI)	Cases in abortions	Abortion Adjusted RR (95% CI)
No pregnancy (reference)	1545	Reference	1545	Reference	1545	Reference
During pregnancy	27	1.17(0.80, 1.72)	11	2.14(1.18, 3.88)	3	0.86(0.28, 2.67)
All follow-up period	1843	1.18(0.99, 1.41)	278	1.18(0.99, 1.42)	226	1.19(1.03, 1.37)
0 to 11 months	204	2.15(1.72, 2.68)	40	1.87(1.33, 2.64)	11	0.75(0.41, 1.35)
12 to 23	149	1.51(1.19, 1.92)	27	1.33(0.89, 1.98)	15	0.99(0.60, 1.65)
24 to 35	121	1.19(0.93, 1.53)	20	1.04(0.66, 1.65)	21	1.40(0.91, 2.17)
36 to 119	721	1.01(0.83, 1.21)	107	1.04(0.82, 1.31)	113	1.23(1.02, 1.49)
120+	648	1.14(0.94, 1.39)	84	1.19(0.92, 1.54)	66	1.23(0.95, 1.58)

Vaginal delivery and CS models were adjusted for age, calendar year, infant sex and place of birth. Abortion models were adjusted for age, calendar year, infant place of birth. We had no information about fetal sex or number of babies in abortions.

**Table 7:** Adjusted relative risk estimates of maternal IBD (Colitis and Crohn's disease) in relation to pregnancy.

Follow-up period	Cases in normal delivery	Normal delivery RR (95% CI)	Cases in CS	CS Adjusted RR (95% CI)	Cases in abortions	Abortion Adjusted RR (95% CI)
No pregnancy (reference)	3442	Reference	3442	Reference	3442	Reference
During pregnancy	26	0.68(0.46, 1.00)	17	2.16(1.34, 3.49)	5	0.56(0.23, 1.35)
All follow-up period	1823	0.91(0.77, 1.07)	263	0.85(0.71, 1.01)	279	0.86(0.76, 0.97)
0 to 11 months	167	1.02(0.82, 1.27)	33	0.98(0.68, 1.41)	16	0.44(0.27, 0.72)
12 to 23	144	0.90(0.71, 1.13)	27	0.87(0.59, 1.30)	26	0.74(0.50, 1.09)
24 to 35	129	0.83(0.65, 1.05)	30	1.06(0.73, 1.55)	31	0.95(0.67, 1.36)
36 to 119	802	0.86(0.72, 1.03)	109	0.79(0.63, 0.99)	156	1.00(0.85, 1.18)
120+	581	0.97(0.81, 1.17)	64	0.81(0.61, 1.07)	50	0.78(0.58, 1.03)

Vaginal delivery and CS models were adjusted for age, calendar year, infant sex and place of birth. Abortion models were adjusted for age, calendar year, infant place of birth. We had no information about fetal sex or number of babies in abortions.

## Discussion

In this study we report the risk of autoimmune disease during and after pregnancy. Overall the risk of AID in women was significantly higher in the first year following vaginal delivery or CS, but was lower in the first year following abortion. While the risk of AID was reduced between the 3rd and 10th year following vaginal delivery, there was no evidence of a change in the risk of AID beyond the first year following CS or abortion. However, women who were pregnant had a higher incidence of AID than those who had no pregnancy records.

Pregnancy has both short and long term effects on the woman's immune system [2]. Fetal microchimerism, or low levels of fetal cells persisting in the mother, is implicated in the pathogenesis of autoimmune diseases, which have a predilection for women after childbearing [18]. While basic molecular studies have implicated persisting fetal cells in the mother in some AID, supportive epidemiological evidence is limited. To our knowledge, this is the first epidemiological study on the risk of AID following pregnancy. The main strength of the paper is the large cohort used and the fact that it is population based, which avoids the problem of selection bias.

We hypothesized that the risk of AID is increased following pregnancy. Further, if fetomaternal cell trafficking is implicated in the etiology of AID after pregnancy, we expected the highest increase in AID diagnosis following (i) CS, due to increased fetomaternal hemorrhage, and (ii) abortion, as fetal loss has been shown to be the only pregnancy complication significantly influencing microchimerism. The first year after pregnancy should be relevant, being the time period closest to the fetomaternal hemorrhage occurring at delivery. Our results confirm an increase in AID in the first year after CS. The unexpected finding of a reduction in AID risk after abortion may be explained by the premise that early fetal loss allows a higher number of fetal stem or progenitor cells to enter maternal blood, and that these cell types are more likely to engraft maternal tissues long-term, and be beneficial in their role [13], [20], [25].

As pregnancy involves complicated and dynamic interactions between the endocrine and immune systems, it is possible that it could have differential effects on the development of an autoimmune disease depending on its timing or even complexity relative to the events that are likely to precede obvious clinical disease [1]. This may explain our findings during and following pregnancy, and the variation between mode of delivery or length of pregnancy.

There were several limitations to our approach. First, since we included data on the first pregnancy only, we did not account for the effect of subsequent pregnancies on the risk of AID. However, the effect we observed on risk of AID was manifest in the first year after pregnancy, which precludes subsequent pregnancies being responsible in the vast majority of women. We accept that in some cases another pregnancy may have occurred in the year follow-up period after a first trimester abortion and that this instead may influence the reduction in AID found. Other studies have previously failed to show an association with parity and the development of AID [1].

Second, it is possible that the increased risk of AID that we observed is linked to the increased risk of AID presenting during pregnancy. Women may be more likely to be tested for AID (along with other diseases) during pregnancy, and it is also possible that some of these diagnoses were not confirmed until after pregnancy. Further, women with symptoms related to AID may experience a complicated pregnancy and thus require delivery by CS. If that is the case then the association that we describe could be related to more testing during pregnancy rather than effects of fetomaternal cell trafficking.

Third, another limitation of the study was the inability to report the risk of AID after spontaneous abortion. This would be relevant as not only are the risks of spontaneous abortion increased with some AID, but also microchimerism is more likely to be established after induced abortion. We were only able to obtain data on induced abortion from the Danish Medical Birth Register.

Finally, we report limited details on the increased risk of each individual AID, as small numbers of affected women in some disease categories during the follow-up period prevents accurate statistical analysis in all groups. Nonetheless we believe these data are important to report, as they show the relationship between pregnancy and maternal AID in a large population-based cohort over a detailed follow-up period.

We suggest an association between pregnancy and the risk of subsequent maternal AID, with a significant impact on the first year after delivery. However, given the number of unanswered questions and the available data, conclusions on the role of fetal microchimerism in the development of autoimmune diseases cannot be drawn.
